# The Combined Effect of High Hydrostatic Pressure and Calcium Salts on the Stability, Solubility and Gel Formation of β-Lactoglobulin

**DOI:** 10.3390/foods4020229

**Published:** 2015-06-08

**Authors:** Daniel Saalfeld, Ina Riegel, Ulrich Kulozik, Ronald Gebhardt

**Affiliations:** 1 Chair for Food Process Engineering and Dairy Technology, Technische Universität München, 85354 Freising-Weihenstephan, Germany; E-Mails: daniel.saalfeld@tum.de (D.S.); ina.riegel@gmail.com (I.R.); 2 Research Centre for Nutrition and Food Science (ZIEL)–Section Technology, Technische Universität München, 85354 Freising-Weihenstephan, Germany; E-Mail: ulrich.kulozik@tum.de

**Keywords:** high hydrostatic pressure, β-Lactoglobulin, Hofmeister series, gel formation

## Abstract

Stability, aggregation and gelation of β-Lactoglobulin are affected by high pressure and salts of the Hofmeister series. Little is known about their combined effects on structure formation processes of β-Lactoglobulin, mainly because many salts of the series are not suitable for use in food. Here, we investigate the effect of calcium salts on the strength of pressure-induced gels, inspired by the fact that high pressure and salts change the water structure in a similar way. We find that the larger the applied pressures, the higher the strength of the gels. In addition to pressure, there is a significant influence by the type of anions and the amount of added calcium salts. Gel strength increases in the order CaCl_2_ < Ca (NO_3_)_2_ < CaI_2_. This trend correlates with the position of the salts in the Hofmeister series. The results are explained by analogy with the thermal aggregate formation by taking reaction rates for unfolding and aggregation, as well as specific/non-specific salts effect into consideration.

## 1. Introduction

In addition to studies concerning its use as a novel preservation technique [1,2], high hydrostatic pressure has been used as a tool to explore stability [3,4], phase transition [5,6] and structure-function-relationships of food macromolecules [7,8]. It has been demonstrated that salt ions in solution induce a similar change in the tetrahedral structure of water as high pressures [9], and it is a challenge to consider this disordering effect for the explanation of unfolding, solubility and precipitation of proteins [10]. In general, anions show more pronounced effects than cations because the former interact more strongly with water at the same size and absolute charge density [11]. Pressure stability of protein assemblies can be increased with the addition of salt.

For instance, it was shown that the pressure dissociation of casein micelles shifted by 200 MPa towards higher pressures after addition of 0.1 M calcium chloride [12,13]. For other processes, pressure can take over the role of salt ions. Instead of cations [14,15], high pressure promotes the conformational transition from random coil to β-sheet, which is necessary for silk fiber formation [16].

In this paper we investigate the combined effect of high hydrostatic pressures and ions of different calcium salts on the strength of β-Lg gels. To induce gel formation, a critical concentration of w/V = 1% is needed [17]. It has been suggested that soluble aggregates act as an intermediate state for the gelation and that disulfide bonds are involved in this process [18]. These aggregates are formed by two successive steps. The first step describes the transformation of native β-Lg into a partially unfolded state. This step determines the overall-rate of the aggregation under conditions where unfolding is slow, for example, at temperatures below T = 85 °C [19] or under salting out-conditions [20]. The second step, the aggregation, determines the overall reaction rate when unfolding is fast. Aggregates can exist either as filaments or particulates, the latter appear optically opaque. Gels formed by filaments have smaller and more homogeneous pore sizes, which cause a better water binding capacity [21]. Particle gels have strong inter-particle bonds and concerning elasticity no polymeric character [22].

Salts influence stability of proteins by changing their solubility. In general, proteins are less stable under conditions where their solubility is high [23]. A decrease in aggregation rate was observed for β-Lg with increasing ionic strength at pH 5.0 [24]. Aggregation of β-Lg is promoted by high pressures at ambient temperatures [25] and *in situ* experiments indicate that aggregates already form under pressure [4]. At high protein concentrations pressure treatments led to β-Lg gelation at pH 7.0 and 25 °C [26]. In this paper we use high hydrostatic pressure treatments and calcium salts to form β-Lg gels at ambient temperatures. For a mechanical characterization of these gels we use force-elongation experiments. We discuss the result on the basis of solubility experiments and basic knowledge about the salt of the Hofmeister series. Our approach aims to gain new knowledge about the gel formation process by taking into account the agonistic effect of high pressure and chaotropic salt on the water structure.

## 2. Experimental Section

### 2.1. Sample Preparation

High purity β-Lg was prepared as described by Toro *et al.* [27]. Protein and salts (CaCl_2_, CaI_2_ (Sigma-Aldrich Chemistry, Steinheim, Germany) CaNO_3_ (Merck KGaA, Darmstadt, Germany) were dissolved in Ultrapure MilliQ water. Final protein concentration (5% w/w) was adjusted by mixing with solutions of concentrated protein and salt. For high pressure treatment samples were mixed in a container (sample volume: 43 mL), vacuumized and double sealed in a polymer foil. Pressurization was done with a pilot pressure-processing unit built by Bolenz & Schäfer (Biedenkopf, Germany). High pressure was varied over a range of 200 to 500 MPa with a constant pressure build up/release rate of 200 MPa/min and a holding time of 2 h at 20 °C for all experiments.

### 2.2. Solubility Experiments

We tested the solubility of β-Lg (native and partially unfolded [8]) with a concentration of 0.1% w/w in each case) in CaCl_2_ solutions (0–4.2 M). After centrifugation at 15,000 g for 45 min we separated the precipitate from the clear supernatant. We estimated the protein concentration by optical density measurements using an absorptivity value of 0.96 L/(g·cm) at 278 nm [28].

### 2.3. HPLC to Determine Degree of Denaturation

The degree of denaturation was investigated by RP-HPLC. Therefore, samples were diluted to reach a protein concentration of 10 gL^−1^ to be within the calibration range. The pH of the dilution was adjusted to 4.6 using 0.1 and 0.01 M HCl or NaOH. Samples were allowed to precipitate and the supernatant was filtered using a syringe filter of 0.45 µm (Chromafil Xtra RC-45/25 Macherey-Nagel, Dueren, Germany). The analysis was performed using an Agilent 1100 series chromatograph (Agilent Technologies, Santa Clara, CA, USA) equipped with a binary pump, column oven, auto-sampler and UV detector. The used column was a PLRP-S 8µ300Å (Latek, Eppelheim, Germany). Elution was performed using a mixture of 57% eluent A (1% Trifluoroacetic acid (TFA) in water) and 43% eluent B (80% Acetonitrile and 0.05% TFA in water) at a flow rate of 1.0 ml min^−1^ at 40 °C. The used gradient of eluent B was as follows: 47% after 2min, 49% after 6 min, 52% after 9 min, 55% after 11.5 min, 100% after 14 min. Total analysis time for each sample was 18 min. The eluent was detected using an UV detector at 226 nm. Ultrapure MilliQ water (MilliQsystem, Millipore, USA), HPLC grade acetonitrile and TFA (Sigma Aldrich Chemistry, Steinheim, Germany) were used to prepare samples and eluents. Peak areas of β-Lg in the HPLC-chromatograms before (A_0_) and after HP treatment (A_p_) were used to calculate the degree of denaturation (DD) according to:

(1) $$\text{DD}=\;\frac{{\text{A}}_{\text{p}}}{{\text{A}}_{0}}\;\cdot 100\%$$

### 2.4. Texture Analysis

Texture analysis was performed using a TA-XT2 (Stable Micro Systems, Godalming, United Kingdom). The sample was penetrated with a cylindrical probe (diameter: 5 mm; height: 20 mm). The sample had a height of at least 25 mm to avoid boundary effects.

## 3. Results and Discussion

**Figure 1:**
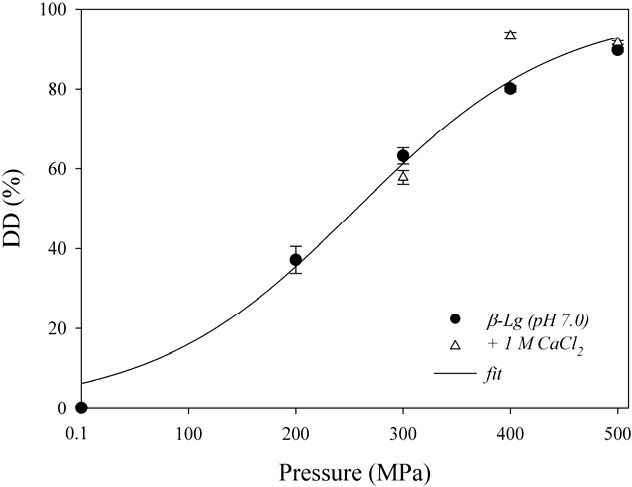
Degree of denaturation, DD, as a function of the hydrostatic pressure applied for treatments of β-Lg without and with 1M CaCl_2_.

We used high-pressure treatments and calcium salts to modulate the structure and self-assembling properties of β-Lg. The set-up allowed only measurements with pressures above 100 MPa. First, we determined the degree of denaturation of β-Lg by HPLC after treatment with different hydrostatic pressures, with and without added salt. Figure 1 shows the degree of denaturation of β-Lg as a function of pressure. The degree of denaturation increased with pressure, as outlined in the literature [3,29]. We used a two-state equilibrium model to analyze the data. The volume change is given by:

(2) $$\text{ΔV}={\left(\frac{\partial \text{ΔG}}{\partial \text{p}}\right)}_{T}=\;-\text{RT }\cdot {\left(\frac{\partial \text{lnK}}{\partial \text{p}}\right)}_{\text{T}}$$

where ΔG is the change in free energy, K the equilibrium constant, R the universal gas constant, p the pressure and T the absolute temperature [30]. From the fit, we estimated an unfolding volume of ΔV = −27 ± 3.1 mL/mol and a free energy of denaturation of ΔG = 6.6 ± 0.8 kJ/mol. These values are in the same range, but significantly smaller, as those reported in the literature [29]. Differences in the values result from the limited sensitivity of the HPLC method. A broader pressure transition for the denaturation resulted compared to more sensitive methods, such as spectroscopic techniques. In the presence of 1 M CaCl_2_, the stability of β-Lg decreased. As a consequence, the degree of denaturation, DD increased from 80.1% ± 0.77% to a maximal value of 93.4% ± 0.89% in the presence of the salt for treatments with 400 MPa.

**Figure 2:**
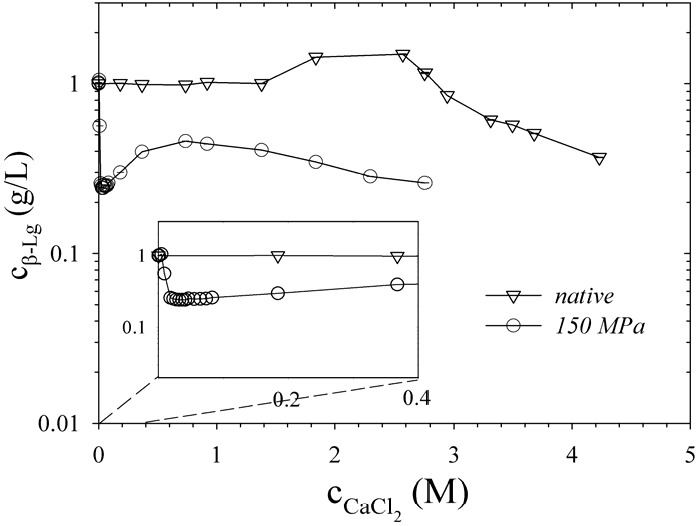
Concentration of untreated and pressurized β-Lg as a function of salt concentration. Calcium chloride was added after high-pressure treatment with 150 MPa.

High-pressure treatments changed the solubility of β-Lg in CaCl_2_ solutions (Figure 2). For solubility experiments we used pressure treatments of 150 MPa. This allowed us to induce unfolding by keeping molecular weight and hydrodynamic radius constant at the same time [8]. Subsequently, calcium chloride was added and precipitates were removed by centrifugation. A salting-in effect can be seen for native β-Lg (without pressure-treatment) up to a concentration of 2.5 M CaCl_2_, followed by salting out at higher molarities. In contrast, an immediate decrease in solubility occurred for pressure-treated β-Lg at the lowest salt concentrations. In a concentration range between 0.2 and 1 M CaCl_2_, an unusual increase in solubility occurred, which was followed again by decrease in solubility. This effect could be caused by ion-induced refolding back into native β-Lg. A reinforcement of the native β-Lg structure by calcium ions has been reported [31].

There was no gel formation of β-Lg at pH 7.0 for all pressure treatments up to 500 MPa. Under these pH conditions, we obtained gels only when calcium salt was added beforehand. However, at pH 4.6 gels formed also in the absence of salt. The missing gel formation at low ionic strength and pH 7 can be well explained by electrostatic repulsion forces, which hinder association. Despite pressure-induced unfolding, one can assume that solubility of β-Lg remains high because of its negative net charge. For pressure treatments up to 250 MPa it was recently demonstrated that molecular weight and hydrodynamic radius of β-Lg remain almost unchanged [8]. However, this aggregation barrier disappears when either the net charge of β-Lg is set close to zero by adjusting the pH near the isoelectric point or the charged proteins are screened by salt. Salts influence also the hydrophobic interactions between proteins in aqueous solution by changing surface tension. The positive surface tension increment of CaCl_2_ [32] results in a decrease in solubility and stronger protein-protein interactions [33].

To characterize the mechanical properties of the resulting β-Lg gels, we used force-elongation experiments.

**Figure 3:**
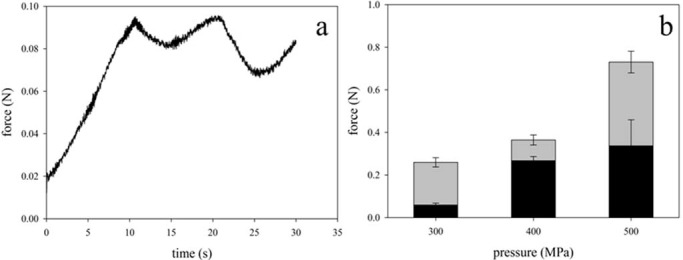
(**a**) Force-elongation curve and (**b**) maximum force for gels formed after treatments with different pressures. Forces for gels of β-Lg at pH 4.6 (black bars) are compared with those formed at pH 7 in the presence of 1 M CaCl_2_ (grey bars).

Figure 3a shows a typical force-elongation curve we obtained for a gel formed after high-pressure treatment at ambient temperature. The modulation in the curve corresponds to breaks in the gel structure. We used the force of the first maximum as a measure of the gel strength. Compared with gels at pH 4.6, strength for gels at pH 7 and 1 M CaCl_2_ was constantly larger for all pressure treatments tested. This can be explained by an increased reactivity of the thiol-groups [34]. Intermolecular disulfide-bonds may significantly contribute to the network at pH 7 and could, hence, be responsible for the higher gel strength. Furthermore, specific binding sites for calcium on the surface of β-Lg were reported [35,36]. Ions, however, rather shield electrostatic repulsion at pH 7 and affect the fraction of bound water instead of being involved in the formation of intermolecular bridges as thermal aggregation experiments suggested [36]. An increasing preferential hydration at higher NaCl concentrations was for instance explained by the binding of electrolytes to β-Lg through dipole-ion interactions [37]. The unusual increase in solubility of pressure treated β-Lg at 1 M CaCl_2_ (compare Figure 2) could be based on similar interactions. In that case, gel strength weakens because the preferential hydration acts against the formation of new intermolecular cross-links [38]. In contrast, it was demonstrated that calcium does not form salt bridges between the proteins at ambient pressure [36]. Such intermolecular bridges are even more unlikely under pressure because of the electrostrictive effect of separate charges [39].

Furthermore, the measured forces were stronger the higher the pressures for the treatments were (Figure 3b). A particularly strong increase occurred after raising the pressure from 400 to 500 MPa. The increase in strength correlates with the higher degree of unfolding (Figure 1). As a result of a higher pressure, equilibrium is shifted towards the unfolded protein. Interconnection of the additional unfolded proteins increases the molecular weight of the aggregates and hence also the gel strength [38]. It is also likely that the rate of aggregation causes different gel strength for these pressure treatments. For a pressure-dependent aggregation, the rate constant k depends on the size and sign of the reaction volume ΔV^≠^ [39] according to:

(3) $${\left(\frac{\partial \text{lnK}}{\partial \text{p}}\right)}_{\text{T}}=\;–\frac{{\text{ΔV}}^{\neq }}{\text{RT}}$$

An increase in pressure would slow down the rate of aggregation under the assumption that the volume of the transition state is larger than that of the partially unfolded state (positive reaction volume ΔV^≠^). The fact that aggregation is generally suppressed under high pressure supports this assumption [40]. Based on these considerations, we conclude that a lower aggregation rate causes more compact aggregates under pressure, which subsequently form harder gels after pressure release.

**Figure 4:**
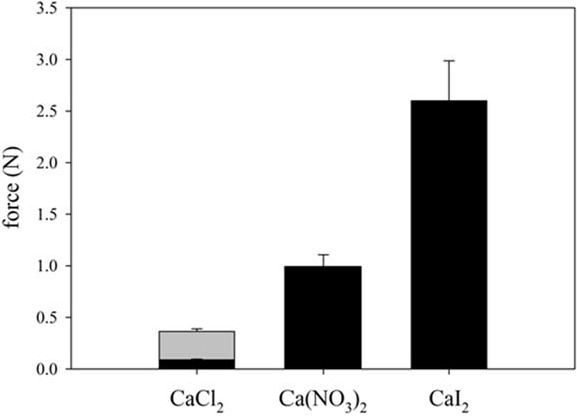
Maximum force of gels obtained after a pressure treatment with 400 MPa in the presence of 100 mM of different calcium salts (black bars). The force obtained for gels with 1M CaCl_2_ is shown again for comparison (grey bar).

We observed a reduction in gel strength by a factor of 4 when we used 100 mM instead of 1 M calcium chloride (Figure 4). At 100 mM almost all negative charges are screened and no further reduction of the repulsion forces at 1 M calcium chloride are to be expected. However, solubility of pressure-treated β-Lg changed in this concentration range. Solubility increased by a factor of 2 after increasing molarity of calcium chloride from 100 mM to 1 M (Figure 2). The increase in solubility causes a decrease in protein stability [23]. As a result, the degree of denaturation reaches its maximum value at 400 MPa (Figure 1). This could change the characteristics of the process from an unfolding-limited to an aggregation-limited reaction as demonstrated for the thermal aggregation process [41]. Pressures ≥400 MPa would accelerate protein denaturation so that aggregation becomes rate-limiting. The harder gels formed after 400 MPa in the presence of 1 M CaCl_2_ are hence a result of the increased availability of unfolded β-Lg and probably of aggregates with a higher molecular weight [38].

Figure 4 further indicates that the type of anions had a significant influence on the gel strength. The strength of the gels increased from Cl^−^ via NO_3_^−^ to I^−^. The force measured by the texture analyzer was more than one order of magnitude higher in the presence of CaI_2_ than with CaCl_2_. It is interesting to note that the gel strength correlates with the position of the salts within the Hofmeister series. As an anion with the highest chaotropic tendency, iodine destabilizes the native structure the most. The same effect can be seen for the cations. Gels with a reduced strength resulted after a pressure treatment with 400 MPa in the presence of 1 M of the less chaotropic sodium and potassium cations (unpublished results). No gel formation took place when only 100 mM of the monovalent cations were added.

**Figure 5:**
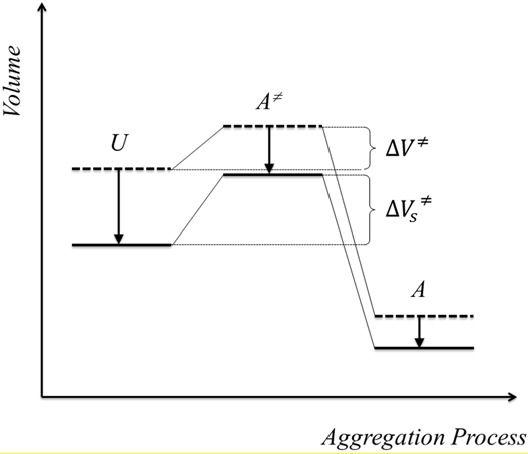
Hypothetical free energy representation of the β-Lg aggregation process under pressure with the volumes of the unfolded (U), transition (A^≠^) and aggregated state (A). Arrows indicate the shift of the states in the presence of chaotropic additives according to [42]. The reaction volumes, ΔV^#^ are also shown.

In the example of the aggregation of lysozyme, it has been shown that in a chaotropic solution the unfolded, transition and aggregated states become all stabilized [42]. A higher energy barrier results, however, because the unfolded state is more stabilized than the transition state. Figure 5 illustrates the situation for an aggregation process under pressure. A salt-induced stabilization of all states would be accompanied by a decrease of their volumes. A volume decrease could, for instance, take place due to protein-bound salt ions. In their vicinity, water molecules are arranged more densely than bulk water due to the electrostrictive effect [43]. Compared to the transition state A^≠^, the salt-induced volume reduction for the unfolded state U would be greater. As a result, there is a deceleration of the aggregation rate k in the presence of salt because ΔV^≠^_S_ > ΔV^≠^ (Equation (3)). Both effects support the argument we already used to explain the formation of harder gel structures. More proteins unfold as soon as the energy level of the unfolded state becomes lower and an overall reaction rate limited by aggregation becomes more probable. The lower aggregation rate could cause denser aggregates, as seen for gels formed after treatment with higher pressures.

## 4. Conclusions

We used high pressure treatment and addition of salt to explore the combined effect on gel formation processes of β-Lg. Hydrostatic pressure and salt equivalently change water structure. However, their impact on protein-protein and protein solvent interaction is diverse and different. Gels with higher strength are formed: (1) the higher the degree of unfolding is and (2) under conditions which decelerate the aggregation rate. The latter is fulfilled when the positive reaction volume of the aggregation process becomes larger, e.g., through stronger chaotropic additives or when a higher pressure is applied. Thus far, our argumentation is based on current knowledge about the effect of salts of the Hofmeister series on the energy state of the thermal aggregation process. In future, reaction rates could be experimentally determined from time-resolved in-situ light scattering experiments, which would help to understand the gel formation process under high pressure in more detail.
